# Lon protease-mediated secretome profiling in *Klebsiella pneumoniae*

**DOI:** 10.1128/mra.00311-25

**Published:** 2026-03-04

**Authors:** Chelsea Reitzel, Jennifer Geddes-McAlister

**Affiliations:** 1 Molecular and Cellular Biology Department, University of Guelph 3653, Guelph, Ontario, Canada; Indiana University, Bloomington, Indiana, USA

**Keywords:** secretome, proteome, protease, *Klebsiella*, iron, pathogenesis, bacteria

## Abstract

Lon protease is a global regulator in bacteria affecting biofilm formation, antibiotic resistance, toxin-antitoxin complexes, and proteolysis of damaged proteins. In *Klebsiella pneumoniae*, Lon has roles in iron response, protein transport, and membrane assembly. Here, we apply bottom-up proteomics to identify proteins in the extracellular environment of WT and Δ*lon K. pneumoniae*.

## ANNOUNCEMENT

In bacteria, Lon protease is a cytosolic enzyme involved in key cellular functions, such as iron homeostasis (1), DNA replication (2–4), virulence (1, 5–7), capsule production (8–10), and stress response, through the proteolysis of damaged proteins. Our previous study on the cellular proteome implications of *lon* deletion in *Klebsiella pneumoniae* highlighted known and novel targets of the protease, including proteins associated with transport (e.g., *PgaA*) and outer membrane assembly (e.g., *BamA*) (1). In this study, we present an investigation into the secretome of Δ*lon* compared to wild-type (WT) *K. pneumoniae* to explore the downstream implications of *lon* disruption on bacterial transport and secretory regulation.

For the experiments, *K. pneumoniae* WT K52 serotype (ATCC 700721) and Δ*lon* were inoculated in 6 mL Luria-Bertani (LB) overnight at 37 °C with 200 RPM shaking. Next, 1 mL of culture was collected, centrifuged at 3,500 *× g* for 10 min, and washed twice with 1 mL M9 minimal media (MM; Chelex 100-treated [Bio-Rad] MilliQ water, glassware rinsed with 3 M HCl, 6.78 g/L Na_2_HPO_4_, 3 g/L KH_2_PO_4_, 0.5 g/L NaCl, 1 g/L NH_4_Cl, and 0.4% [w/v] glucose with 2 mM MgSO_4_ and 0.1 mM CaCl_2_) (1). Cells were resuspended in 1 mL MM and subcultured into 5 mL of LB (i.e*.*, rich), MM (i.e*.*, iron limited), or MM supplemented with 10 µM Fe_2_(SO_4_)_3_ (i.e., iron replete). Cells were grown at 37 °C with 200 RPM shaking, and the supernatant was collected at late-log phase (approximately 14 h for strains grown in LB and 6 h for strains in MM).

Samples were prepared for mass spectrometry analysis as previously described (1, 11–14), with five replicates per strain and media condition. Briefly, 1.2 mL supernatant was filtered using a 0.22 µm pore filter syringe; 333 µL of 8 M urea/40 mM HEPES was added; and samples were reduced using dithiothreitol (10 mM final concentration) at 95 °C for 10 min (800 rpm) and alkylated using iodoacetamide for 20 min (55 mM final concentration; room temperature, in the dark). Samples were digested overnight at room temperature with 4 µL trypsin/LysC protease (Promega). Peptides were dried and purified using STop And Go (STAGE) tips (15) and reconstituted in 40 µL 0.1% formic acid. Next, 1.5 µg of each sample (according to Thermo NanoDrop One measurement at 280 nm wavelength) was loaded onto Evotips according to the manufacturer’s instructions (16) and analyzed with a 15 cm PepSep column (150 µm diameter and 1.9 µM beads) over 88 min on an Evosep One liquid chromatography system coupled to a Thermo Scientific Orbitrap Exploris 240. Mass spectrometry analysis was performed in data-dependent, positive ion mode with 400–2,000 *m*/*z* precursor range, 60,000 resolution, and intensity threshold of 2.5e4. Charge states 2–8 were included.

The .RAW files were processed using MaxQuant v2.4.0.0 (17) with default parameters unless stated otherwise. Peptides were searched against *K. pneumoniae* subsp. *pneumoniae* K52 serotype (5,126 protein sequences from UP000000265 proteome ID; accessed 2 Dec. 2022) (18). Modifications: trypsin specificity with a maximum of two missed cleavages, cysteine carbamidomethylation (fixed), and methionine oxidation and N-acetylation (variable). The number of peptides for protein identification was two, and protein abundance was quantified by label-free quantification (LFQ) with a ratio count set to 1 (19). Match between runs was enabled with a match window of 0.7 min and an alignment window of 20 min (19).

In Perseus v2.0.7.0 (20), contaminants modified by site and reverse peptides were removed, and valid value filtering of three out of five replicates per media condition was set. LFQ intensities were log_2_-transformed, and missing values were replaced by imputation from the normal distribution (i.e., by downshift of −1.8 standard deviations from the mean, with values selected within a 0.3 standard deviation width; between −1.95 and −1.65 standard deviation window). Significant differences in protein abundance were determined by a Student’s *t*-test using a confidence interval of 95%, Benjamini–Hochberg false discovery rate (FDR) cutoff of 5%, and an *S*_0_ = 1. Visualization performed using ProteoPlotter (21).

**Fig 1:**
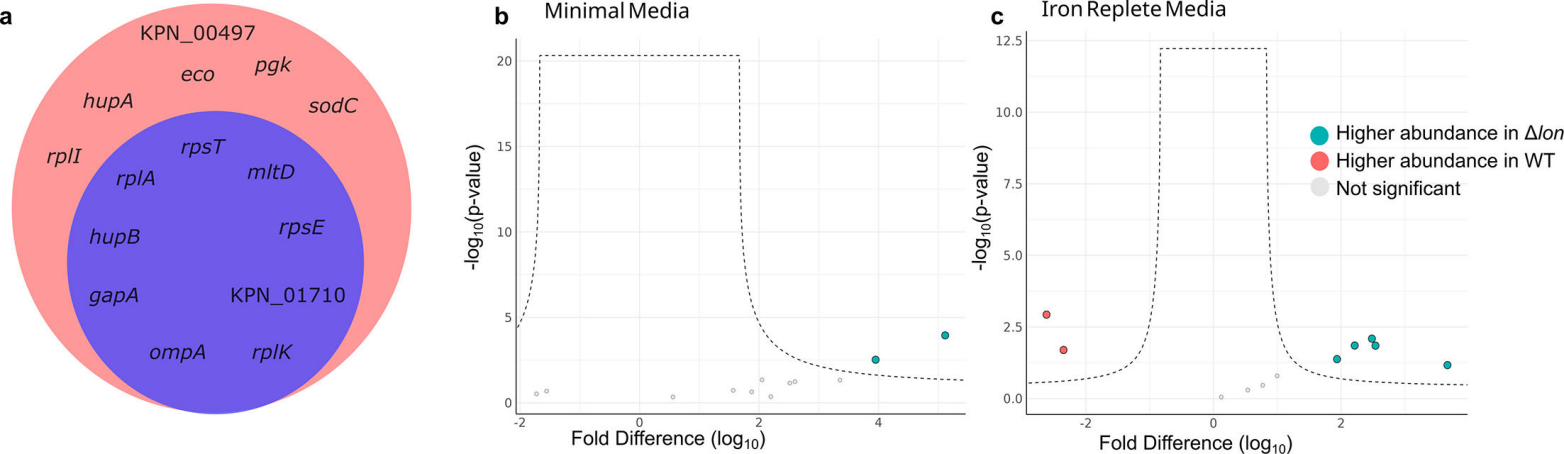
WT and Δ*lon* secretome profiling. (**a**) Venn diagram illustrating identification of proteins in WT and Δ*lon* secretome. (**b**) Volcano plot illustrating proteins more abundant in Δ*lon* versus WT secretome in LIM condition. (**c**) Volcano plot illustrating proteins more abundant in Δ*lon* versus WT secretome in iron-replete condition. Statistical analysis for volcano plot is a Student’s *t*-test, *P*-value < 0.05; FDR = 0.05; *S*_0_ = 1.

We identified 15 proteins secreted by Δ*lon* and nine proteins secreted by WT (across all three media conditions) (Table 1); six proteins were exclusive to the Δ*lon* secretome (Fig. 1a). Notably, ribosomal proteins were detected in the secretome, suggesting potential cell lysis or identification of extracellular vesicle-associated proteins (22). Two proteins were identified in the Δ*lon* secretome in higher abundance than WT in LIM (i.e., RpsT, RplK; Fig. 1b); five proteins were more abundant in Δ*lon* compared to WT in iron-replete conditions (i.e., HupB, RplI, RplA, GapA, and HupA; Fig. 1c), and three proteins were more abundant in WT compared to Δ*lon* in iron-replete conditions (i.e., RplK, RpsT, and MltD; Fig. 1c).

**TABLE 1:** Proteins identified in Δ*lon* and WT secretomes in LB, iron replete, or minimal media and their associated function based on UniProt annotations (23)

UniProt accession no.	Gene name	Media condition	Strain	Protein function
A6T4F8	*rpsT*	Iron replete and LIM	Δ*lon* & WT	30S ribosomal protein S20
A6T509	*mltD*	Iron replete and LIM	Δ*lon* & WT	Peptidoglycan lytic exotransglycosylase
A6T5I3	*hupB*	Iron replete and LIM	Δ*lon* & WT	DNA-binding protein HU-beta, NS1 (HU-1)
A6T5S6	KPN_00497	LB	Δ*lon*	YdgH/BhsA/McbA-like domain-containing protein
A6T751	*ompA*	Iron replete and LIM	Δ*lon* & WT	Outer membrane protein A
A6T7Q9	*gapA*	Iron replete and LIM	Δ*lon* & WT	Glyceraldehyde-3-phosphate dehydrogenase
A6T970	KPN_01710	Iron replete and LIM	Δ*lon* & WT	Glyceraldehyde-3-phosphate dehydrogenase
A6T9Z5	*sodC*	LB	Δ*lon*	Superoxide dismutase [Cu-Zn]
A6TBS4	*eco*	LB	Δ*lon*	Ecotin
A6TDT1	*pgk*	LIM	Δ*lon*	Phosphoglycerate kinase
A6TEV5	*rpsE*	Iron replete and LIM	Δ*lon* & WT	Small ribosomal subunit protein uS5
A6TGN5	*rplK*	Iron replete and LIM	Δ*lon* & WT	50S ribosomal protein L11
A6TGN6	*rplA*	Iron replete and LIM	Δ*lon* & WT	50S ribosomal protein L1
A6TGQ7	*hupA*	Iron replete and LIM	Δ*lon*	DNA-binding protein HU-alpha
A6THB4	*rplI*	Iron replete and LIM	Δ*lon*	50S ribosomal protein L9

## Data Availability

The mass spectrometry proteomics data have been deposited to the ProteomeXchange Consortium via the PRIDE partner repository with the dataset identifier PXD057415.
